# Supplementary material for: Protein Complexes Are under Evolutionary Selection to Assemble via Ordered Pathways
Source: Cell. 2013 Apr 11;153(2):461–70. doi: 10.1016/j.cell.2013.02.044 (PMC4009401; doi:10.1016/j.cell.2013.02.044)
Supplement: Document S1. Article plus Supplemental Information [file mmc5.pdf]

Cell

Protein Complexes Are under Evolutionary Selection to Assemble via Ordered Pathways

Joseph A. Marsh,[1] Helena Hernández,[2] Zoe Hall,[2] Sebastian E. Ahnert,[3] Tina Perica,[4] Carol V. Robinson,[2,*] and Sarah A. Teichmann[1,5,*]
[1]EMBL-European Bioinformatics Institute, Wellcome Trust Genome Campus, Hinxton, Cambridge CB10 1SD, UK
[2]Physical and Theoretical Chemistry Laboratory, Department of Chemistry, University of Oxford, South Parks Road, Oxford OX1 3QZ, UK
[3]Theory of Condensed Matter, Cavendish Laboratory, JJ Thomson Avenue, Cambridge CB3 0HE, UK
[4]MRC Laboratory of Molecular Biology, Hills Road, Cambridge CB2 0QH, UK
[5]Wellcome Trust Sanger Institute, Wellcome Trust Genome Campus, Hinxton, Cambridge CB10 1SA, UK
*Correspondence: carol.robinson@chem.ox.ac.uk (C.V.R.), sarah.teichmann@ebi.ac.uk (S.A.T.)
http://dx.doi.org/10.1016/j.cell.2013.02.044

SUMMARY

Is the order in which proteins assemble into complexes important for biological function? Here, we seek to address this by searching for evidence of evolutionary selection for ordered protein complex assembly. First, we experimentally characterize the assembly pathways of several heteromeric complexes and show that they can be simply predicted from their three-dimensional structures. Then, by mapping gene fusion events identified from fully sequenced genomes onto protein complex assembly pathways, we demonstrate evolutionary selection for conservation of assembly order. Furthermore, using structural and high-throughput interaction data, we show that fusion tends to optimize assembly by simplifying protein complex topologies. Finally, we observe protein structural constraints on the gene order of fusion that impact the potential for fusion to affect assembly. Together, these results reveal the intimate relationships among protein assembly, quaternary structure, and evolution and demonstrate on a genome-wide scale the biological importance of ordered assembly pathways.

INTRODUCTION

In order to function, most proteins assemble into complexes—either homomers, comprised of self-interacting copies of a single type of subunit, or heteromers, composed of two or more distinct polypeptide chains. Is the order in which protein subunits associate important for the formation and biological function of the final complex? Although protein interactions have been studied extensively (Janin et al., 2007; Shoemaker and Panchenko, 2007) and the misassembly of proteins can have severe biological consequences (Dobson, 2003; Ellis, 2007), the multistep process by which proteins assemble into complexes has received comparatively little attention in recent years. By analogy to Levinthal's paradox of protein folding (Levinthal, 1969), we can presume that assembly must proceed via energetically favorable intermediate subcomplexes, lest the time required for productive multisubunit complex formation be prohibitively long. Thus, just as proteins preferentially fold via a limited number of energetically favorable folding pathways (Lindorff-Larsen et al., 2011), protein complexes should be expected to assemble via ordered assembly pathways.

Ordered assembly has now been observed experimentally for a number of systems. Classic studies used a variety of techniques to characterize putative assembly intermediates, which in combination with kinetic measurements, allowed the assembly of various homomeric and heteromeric complexes to be characterized (Friedman and Beychok, 1979). In addition, ordered assembly has been seen in larger multisubunit complexes such as the spliceosomal snRNP core (Raker et al., 1996), the preinitiation transcription complex (Baldick et al., 1994), and the 26S proteasome (Gallastegui and Groll, 2010). In recent years, electrospray mass spectrometry (MS) has emerged as an extremely useful method for studying assembly, having the distinct advantage of being able to probe the oligomeric states of multiple subcomplex intermediates simultaneously, thus allowing in vitro ordered assembly pathways to be elucidated in detail (Sobott et al., 2002; Hernández and Robinson, 2007; Levy et al., 2008).

A powerful way to demonstrate the importance of assembly order would be to test whether assembly pathways have been conserved in evolution. A large-scale analysis of simple homomeric complexes suggested that the order of self-assembly for identical subunits recapitulates quaternary structure evolution and is generally conserved (Levy et al., 2008). However, in heteromers, which account for most in vivo protein complexes (Kühner et al., 2009), the relationship between assembly and evolution has not been investigated. Since there are far fewer published structures for heteromers than for homomers (Perica et al., 2012), it is difficult to employ a similar strategy. Fortunately,

CrossMark

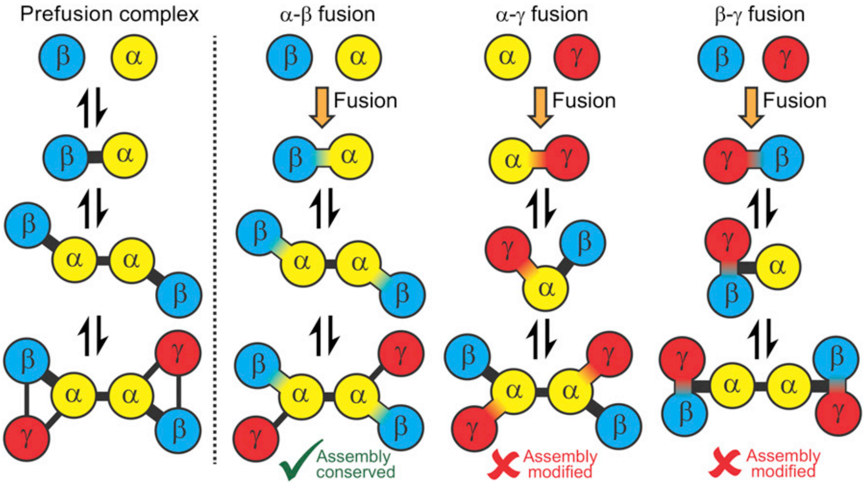

Figure 1. Gene Fusion Events between the Subunits of Protein Complexes Can Either Conserve or Modify Assembly Pathways

This diagram demonstrates the three possible fusion events that could occur in a complex with three unique subunits, each repeated twice. With the α-β fusion, the fusion event mimics the first step of assembly, and thus the assembly pathway would be conserved. However, for both the α-γ and β-γ fusions, the assembly pathway is modified. In this graph representation of protein complexes, a circular node represents each protein subunit, and the edges between nodes represent the intersubunit interfaces.

however, we have identified a unique evolutionary phenomenon that allows us to test whether heteromer assembly pathways have been conserved: gene fusion.

Gene fusion occurs when two previously distinct genes become fused into a single open reading frame. A considerable number of studies have focused on understanding gene fusion as an evolutionary mechanism at the DNA sequence and protein domain levels. In fact, evolutionary reconstructions suggest that gene fusion is the most common mechanism by which multidomain proteins acquire new domains in both bacteria and higher eukaryotes (Björklund et al., 2005; Pasek et al., 2006; Buljan et al., 2010). Gene fusion has received extensive attention since it was shown that evolutionary fusion events could be used to predict protein interactions on a genomic scale (Enright et al., 1999; Marcotte et al., 1999a, 1999b). Essentially, the idea is that proteins that are encoded by different genes in one organism but fused together in another are very likely to physically interact, or at least be functionally related, when expressed as separate gene products. This has been supported by comprehensive analyses (Enright and Ouzounis, 2001; Yanai et al., 2001; Marcotte and Marcotte, 2002; Kamburov et al., 2007; Reid et al., 2010).

Because gene fusion forces the permanent, covalent association of two protein subunits, it provides a mechanism by which protein complex assembly pathways can be either conserved or modified in evolution. As illustrated in Figure 1, a fusion event can be compatible with and conserve the existing assembly pathway if it mimics the first step of assembly. Alternatively, a fusion-induced linkage can disrupt the order of assembly. Therefore, if careful examination of the evolutionary record were to reveal a significant tendency for gene fusion events that conserve rather than modify existing protein-complex assembly pathways, this would strongly support the importance of ordered assembly for the formation of functional protein complexes.

Here, we exploit the large number of fully sequenced genomes and protein complex structures that are now available in order to identify evolutionary gene fusion events that have occurred between genes encoding the subunits of heteromeric complexes. First, by experimentally determining the assembly pathways of several of these complexes, we show that assembly can be predicted on a large scale from crystal structures. This allows us to demonstrate significant evolutionary selection for gene fusion events that conserve the existing order of subunit assembly. In addition, we observe a tendency for fusion to optimize assembly by maximally reducing the interfaces in protein complexes and discrete interactions in protein interaction networks. Finally, we show protein structural constraints on the gene order of fusion, which arise from a preference for optimally positioned N and C termini and influence the potential for fusion to affect assembly. Overall, these results demonstrate the role of protein complex assembly in evolution and provide fundamental insight into the biophysics and biological importance of ordered assembly pathways.

RESULTS

Prediction of Heteromer Assembly Pathways and Characterization by Nanoelectrospray Ionization MS

We first searched the Protein Data Bank (Berman et al., 2000) for heteromeric complexes for which there is genomic evidence of fusion occurring between subunits in the STRING database (Szklarczyk et al., 2011). In each of these complexes, a pair of subunits is encoded by two separate genes that are known to become fused in another species. We refer to these as "prefusion" complexes because they are likely to be similar to the ancestral complexes that existed prior to the evolutionary gene fusion event. In total, we identified 94 nonredundant pairs of heteromeric subunits associated with fusion events (Table S1). Thus, if we knew the assembly pathways of these complexes, we could assess whether the evolutionary fusion events were compatible with the existing order of assembly and would have conserved that order.

Previously, we showed that one can predict the assembly of homomeric complexes by invoking a simple model in which the strength of each interface is assumed to be proportional to the surface area buried between the two subunits, as calculated from the crystal structure (Levy et al., 2008). However, we were uncertain whether a similar phenomenon would hold true for heteromeric complexes, especially considering that interface size generally shows weak correlation with binding affinity in heteromers (Brooijmans et al., 2002), and that heteromeric subunits are

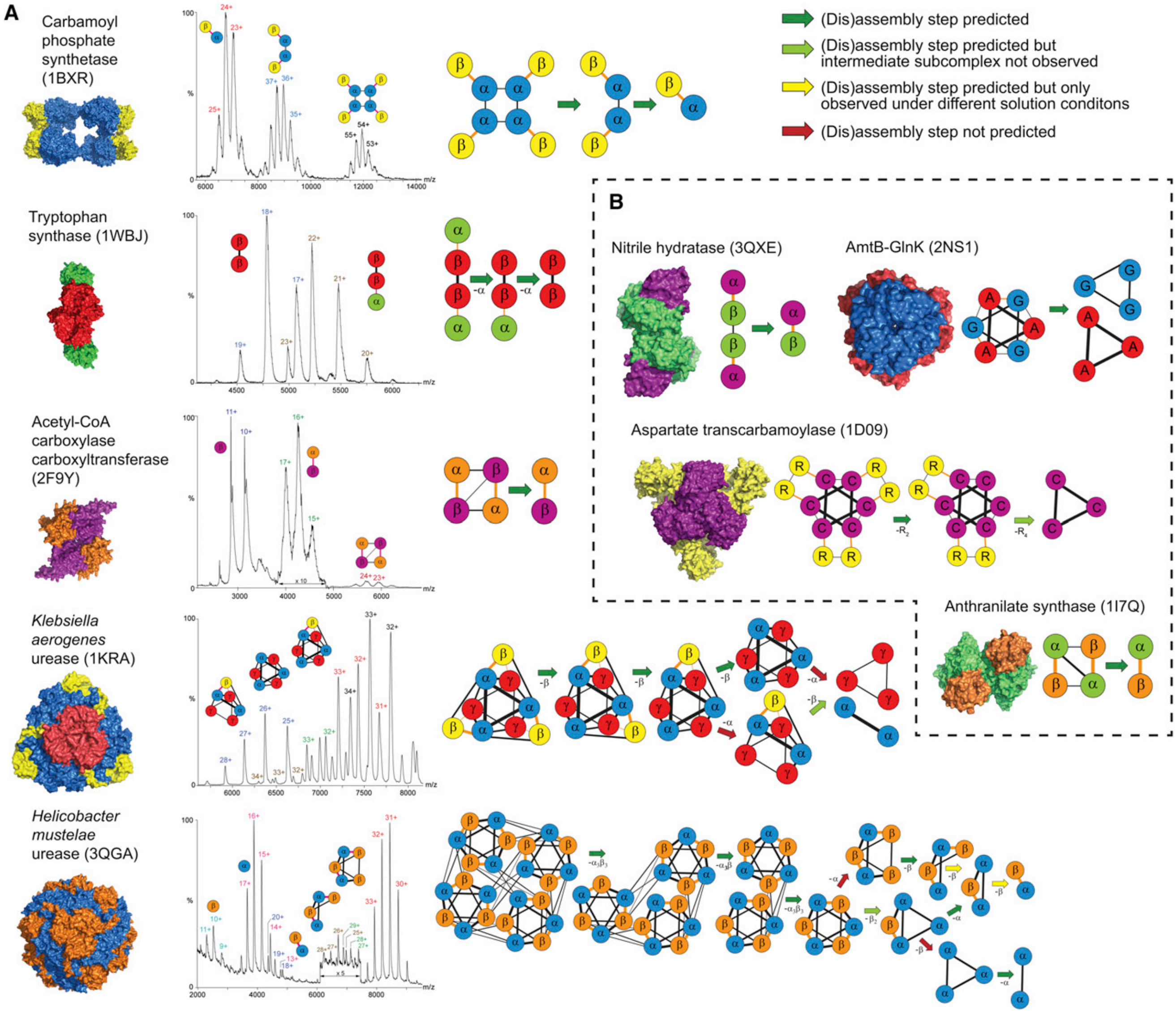

Figure 2. Experimentally Characterized (Dis)Assembly Pathways of Heteromeric Prefusion Complexes

(A) (Dis)assembly pathways of complexes characterized by nESI-MS as well as representative mass spectra. See Table S2 for a full list of subcomplexes identified under different solution conditions.

(B) (Dis)assembly pathways of complexes identified from previously published experiments. In the graph representations of protein complexes, interfaces that undergo fusion are shown in orange.

See also Figure S1.

often more flexible in isolation and tend to undergo larger conformational changes upon binding (Marsh and Teichmann, 2011; Marsh et al., 2012). Furthermore, the presence of multiple distinct subunits means that heteromers have far more potential routes of assembly, which could complicate predictions.

To test the association between interface size and assembly, we performed nanoelectrospray ionization (nESI)-MS experiments (Sobott et al., 2002; Hernández and Robinson, 2007) on five of the prefusion complexes identified above in order to determine their reversible in vitro disassembly pathways. Representative mass spectra are shown in Figures 2A and S1. Although the process of disassembly is different from that of assembly, the two processes are generally reversible in homomeric complexes (Levy et al., 2008). To further support this notion, we show that the prefusion complexes studied here can be reassembled from their dissociated states without the formation of off-pathway subcomplexes, thus demonstrating the reversibility of assembly and disassembly in heteromers (Figure S2). Therefore, we refer to "(dis)assembly" as this reversible process we can probe in solution.

In addition to the MS experiments, we also identified four prefusion complexes in which (dis)assembly pathways could be

Table 1. Heteromeric Prefusion Complexes of Known Structure with Experimentally Characterized (Dis)Assembly Pathways and Their Agreement with Prediction

Complex Name	PDB ID	Correctly Predicted Steps
Carbamoyl phosphate synthase	1BXR	2/2
Tryptophan synthase	1WBJ	2/2
Acetyl-CoA carboxylase carboxyltransferase	2F9Y	1/1
Klebsiella aerogenes urease	1KRA	4/6
Helicobacter mustelae urease	3QGA	9/11
Nitrile hydratase	3QXE	1/1
AmtB-GlnK	2NS1	1/1
Aspartate transcarbamoylase	1D09	2/2
Anthranilate synthase	2NS1	1/1
Total		23/27

See also Figure S2.

inferred from previously published literature (Evans et al., 1974; Poulsen et al., 1993; Payne et al., 1997; Durand and Merrick, 2006). The full (dis)assembly pathways for all nine complexes are shown in Figure 2 and detailed descriptions are provided in the Extended Experimental Procedures. We found excellent agreement between interface sizes and (dis)assembly, with seven out of nine complexes (23/27 total steps) agreeing perfectly with predictions (Table 1). This strongly demonstrates that the (dis)assembly of both homomeric and heteromeric complexes is primarily determined by the sizes of their interfaces and can therefore be easily predicted.

It is interesting to note the two complexes that show some deviations from the assembly predictions. These are both related urease complexes, representing two separate fusion events. In each case, the first few (dis)assembly steps proceed exactly as predicted, followed by a split into parallel pathways that is not predicted. We hypothesize that, for these large complexes, the loss of some subunits may lead to tertiary and/or quaternary structural rearrangements, which could change the relative interface sizes. Thus, the interface model might still hold in these cases, if only we knew the conformational rearrangements that occur upon subunit loss.

Evolutionary Selection for Conservation of Protein Complex Assembly Pathways upon Gene Fusion

The ability to confidently predict (dis)assembly from crystal structures enables us to simulate (dis)assembly pathways on a large scale for all protein complexes of known structure. We can then investigate in detail the tendency for assembly to be conserved or modified by the 94 nonredundant evolutionary gene fusion events associated with prefusion complexes.

We first considered the intrinsic likelihood of subunit fusions either conserving or modifying (dis)assembly pathways. For each heteromeric pair of subunits in a large set of nonredundant complexes, we assessed the effects of a hypothetical fusion event on the (dis)assembly pathway, regardless of whether or not there was actually any genomic evidence for fusion occurring between them. Of the 1,487 hypothetical fusions that could occur between nonredundant subunit pairs, only 201 (13.5%) would conserve (dis)assembly, and the remainder would disrupt existing (dis)assembly pathways (Figure 3A). Thus, we can immediately see that if fusion were to occur randomly between the subunits of heteromeric complexes (i.e., without evolutionary selection), assembly-conserving fusion events would be quite rare.

Next we looked at how frequently actual evolutionary gene fusion events have occurred in these two groups. Whereas 24/201 (11.9%) subunit pairs that would conserve (dis)assembly pathways actually have evolutionary evidence for fusion occurring between them in some other species, this is true for only 48/1,286 (3.7%) pairs that would modify (dis)assembly ($p = 8 \times 10^{-6}$, Fisher's exact test; Figure 3A). Thus, although the large majority of heteromeric subunit pairs show no evidence of fusion, a fusion event is far more likely to occur if it conserves the existing assembly pathway.

An alternate means of testing for assembly conservation is to compare the frequency with which (dis)assembly pathways are conserved in our set of evolutionary gene fusion events with the frequency we would expect based upon the intrinsic topologies of the complexes. We implemented a simple null model in which the quaternary structure topology of each complex was retained but random weights were assigned to each unique interface type. We then predicted the (dis)assembly pathway for each randomly reweighted complex and assessed the conservation frequency, and repeated this process many times in order to calculate the intrinsic probability of assembly conservation. The observed frequency with which real evolutionary gene fusion events conserve (dis)assembly is 33.3% (24/72), which is nearly double the intrinsic expectation for complexes with the same topologies according to this model (17.3%, $p = 1 \times 10^{-4}$; Figure 3B). In fact, a marginal level of significance is retained even when only the nine experimentally characterized complexes are considered (44.4% [4/9] conserved versus 19.5% expected [$p = 0.05$]).

Finally, to investigate the evolutionary selection for assembly-conserving gene fusion events more directly, we considered only heteromeric complexes with more than two unique subunits. In these complexes, multiple fusion events are hypothetically possible, which allows us to assess the probability of assembly conservation if fusion occurred randomly (e.g., for a complex with three unique subunits, as in Figure 1, each fusion would have a one in three chance of occurring). We observe that 38.9% (14/36) evolutionary fusion events in these complexes conserve (dis)assembly, compared with only 14.9% expected if fusions had randomly occurred within the same complexes ($p = 7 \times 10^{-5}$; Figure 3C). Therefore, given the set of fusion events that are hypothetically possible within a heteromeric complex, evolution appears to have strongly preferred those that mimic and thus conserve existing assembly pathways.

Above we have shown that (dis)assembly in heteromers is primarily driven by the sizes of the intersubunit interfaces. Large interfaces have been noted as characteristic of obligate complexes, in which the subunits are permanently associated within the cell (Nooren and Thornton, 2003). In Figure S3 and the Extended Experimental Procedures, we present multiple

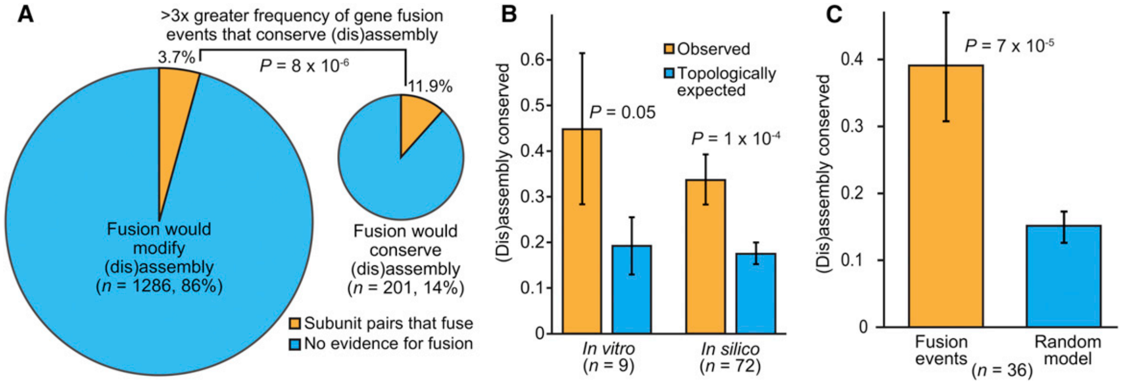

Figure 3. Evolutionary Conservation of Protein Complex (Dis)Assembly Pathways upon Gene Fusion

(A) Comparison of the frequency of evolutionary gene fusion events in heteromeric subunits pairs that would either conserve or modify (dis)assembly pathways upon hypothetical subunit fusion.

(B) Comparison of observed (dis)assembly conservation from in vitro experiments and in silico predictions with the intrinsically expected values for complexes with the same topologies.

(C) Direct comparison of predicted (dis)assembly conservation and randomly occurring fusions in complexes with more than two unique subunits. Error bars represent the SEM.

See also Figure S3 and Table S1.

lines of evidence that fusion occurs preferentially in obligate complexes, including a lower tendency for fusing subunits to be observed in isolation and a much higher propensity for correlated messenger RNA (mRNA) expression. Importantly, we show that the observed assembly conservation does not arise from a tendency for fusion to occur in obligate complexes.

Taken together, our results provide robust evidence of evolutionary selection for assembly-conserving gene fusion events. Importantly, we emphasize that this is not an absolute rule, and that a slight majority of fusions do in fact disrupt assembly. However, one must consider that random subunit fusions would conserve (dis)assembly in only a very small fraction of cases and thus the evolutionary frequency of (dis)assembly-conserving fusions is far higher than would be expected by chance.

Optimization of Assembly upon Fusion through Simplification of Protein Complex Topologies

Despite the strong selection for assembly conservation, it is clear that many evolutionary fusion events have modulated existing assembly pathways. Thus, we hypothesized that there may have been further evolutionary selection for fusion events that optimize assembly. For instance, although any fusion event between subunits will reduce the number of assembly steps by at least one, greater simplification will occur if the fusion involves two subunits that both share other interaction partners, as this will result in fewer intermolecular interfaces in the fused complex (Figure 4A).

We compared the reduction of intersubunit interfaces in protein complexes upon fusion with what would be expected if fusion occurred randomly between subunits (essentially as in Figure 3C). Interestingly, we observed that gene fusion events tended to reduce the number of interfaces by considerably more than would be expected by chance (2.90 versus 2.21, $p = 1 \times 10^{-4}$; Figure 4B). This strongly implies evolutionary selection for fusions that maximally reduce the number of interfaces in a protein complex, thereby simplifying their topologies and assembly pathways. We suggest that having fewer intersubunit interfaces would both lower the risk of misassembly and increase the speed of assembly.

We investigated this phenomenon further by searching high-throughput interaction data for interacting proteins with evidence of fusion occurring between them. Each binding partner shared by a pair of proteins will further reduce the number of distinct protein-protein interactions by one upon fusion (Figure 4C). Pairs of proteins from *Escherichia coli* that undergo fusion share a mean of 19.2% of their binding partners, compared with 13.2% expected for random fusions within the interaction network ($p = 3 \times 10^{-4}$; Figure 4D). Similar trends are also seen in yeast (14.7% versus 7.1%, p = 0.008), humans (23.2% versus 16.4%, p = 0.04), and a large number of other species (Table S3). Contrary to our structure-based analysis, if two proteins share a binding partner in these high-throughput data, it does not necessarily mean that they are interacting simultaneously (Kim et al., 2006a). Nevertheless, these results imply evolutionary selection for fusion events that optimize network topology by reducing the number of discrete protein interactions, in analogy to the simplification of assembly.

Protein Structural Constraints on Fusion

Because gene fusion essentially forces a pair of proteins to interact permanently with each other, the influence of fusion on assembly may be limited by protein structural constraints dictating whether or not a fusion event is likely to occur. Upon fusion of two proteins, the C terminus of the first will become covalently linked to the N terminus of the second. If these termini are far apart in the prefusion complex, fusion would require either the addition of a lengthy linker or a major disruption of the intersubunit interface. However, if these termini are close in space, fusion would be more likely to conserve the existing quaternary structure (Figure 5A).

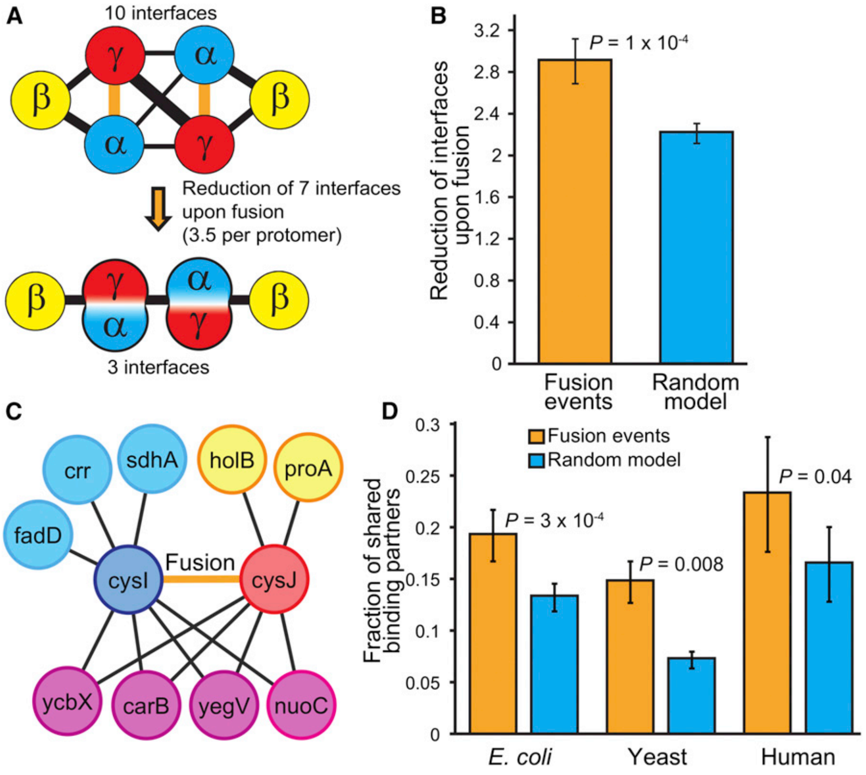

Figure 4. Evolutionary Simplification of Protein Complex Assembly via Gene Fusion

(A) Graph representation of a prefusion complex (PDB ID: 1RM6) in which the subunits that fuse (α and γ) share interaction partners, leading to a large decrease in the number of interfaces upon fusion.

(B) Mean reduction in interfaces (per protomer) upon fusion for 36 fusion events, compared with random fusions within the same complexes.

(C) Protein-protein interaction network for the *E. coli* proteins cysI and cysJ showing that four out of nine binding partners (magenta) are shared between the two; thus, the total number of discrete interactions will be reduced by four upon fusion.

(D) Comparison of shared binding partners between proteins that undergo fusion from high-throughput protein interaction data for *E. coli* (n = 61), yeast (n = 16), and humans (n = 16). Comparisons for 411 other species are provided in Table S3. Error bars represent the SEM.

See also Table S1.

To illustrate this, we consider the case of the prefusion complex *Klebsiella aerogenes* urease (Jabri and Karplus, 1996), where fusion is known to occur between genes corresponding to the γ and β subunits. Because the γ subunit fuses upstream of the β subunit, fusion will result in a linkage between the C terminus of the γ subunit and the N terminus of the β subunit. Examination of the complex crystal structure reveals that these termini are in fact quite close, separated by only 16 Å (Figure 5B). We will refer to this as the "fusion distance." The "reverse distance" (if fusion were to occur in the opposite gene order [i.e., β upstream of γ]) is much greater (66 Å).

We systematically compared the fusion and reverse distances of all prefusion complexes in our data set in which the subunits correspond closely to the full-length genes (Figure 5C). We observe that for cases in which fusion has occurred in only a single gene order, the fusion distances are shorter than the reverse distances in 35/47 (74.5%) fusion events ($p = 0.001$, binomial test). Furthermore, the mean fusion distance is 14.1 Å shorter than the mean reverse distance ($p = 0.001$, Wilcoxon signed-rank test). Importantly, this tendency for fusion to occur between the closer termini is not related to the (dis)assembly conservation demonstrated earlier (see Extended Experimental Procedures). Therefore, the order of gene fusion is closely related to the structure of protein complexes, with significant evolutionary selection for fusion events that link more proximal termini. This is consistent with a previous study in which pairs of domains that were observed to interact both inter- and intramolecularly, which included several fusions, were shown to conserve their binding orientations in most cases (Kim et al., 2006b).

DISCUSSION

By comparing the identities of assembly intermediates observed in nESI-MS experiments with the structures of protein complexes, we were able to gain a fundamental mechanistic insight into protein assembly. Essentially, assembly in both homomeric and heteromeric complexes is driven by the hierarchy of interface sizes within a protein complex, such that assembly intermediates will tend to possess larger intersubunit interfaces. By taking advantage of Nature's grand protein engineering experiment, i.e., the large number of gene fusion events that have occurred throughout evolutionary history, we show that these assembly intermediates are under evolutionary selection. This suggests that modifying existing assembly pathways has a significant tendency to lower an organism's evolutionary fitness.

Although numerous functional benefits arise from the formation of multisubunit complexes, the increased complexity is associated with a greater risk of misassembly. Our results suggest that evolution has selected for protein complexes that assemble via well-defined, ordered pathways. Presumably, this leads to faster and more efficient formation of the functional complexes. If these assembly pathways become modified in evolution, the identities of the assembly intermediates will change, potentially increasing their susceptibility to misassembly or aggregation. Thus, the evolutionary conservation and optimization of assembly pathways revealed here provide a potential means of minimizing these risks while maintaining the advantages of complex formation. Furthermore, our results have practical implications in that the identities of assembly intermediates can now be predicted from the three-dimensional structures of protein complexes. This may provide clues as to how misassembly occurs and how it might be prevented.

The assembly and quaternary structure of protein complexes are highly important for determining which gene fusion events

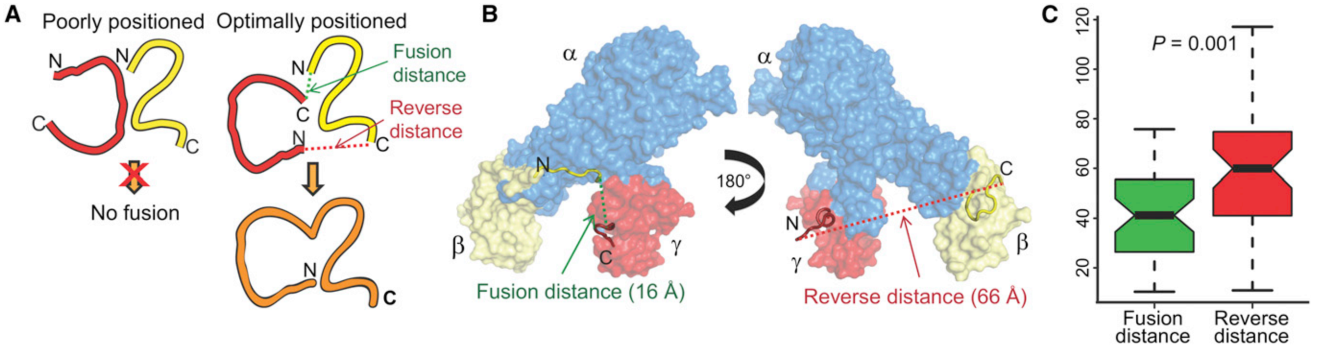

Figure 5. Protein Structural Determinants of Gene Fusion

(A) Fusion may be unable to occur if the protein termini are too far apart in the prefusion complex. However, if the C terminus of one subunit is close to the N terminus of the other, a productive fusion is more likely.

(B) Comparison of fusion and reverse distances between the γ and β subunits of *K. aerogenes* urease (PDB ID: 1KRA; only one $\alpha\beta\gamma$ trimer from the full $(\alpha\beta\gamma)_3$ nonamer is shown).

(C) Box plot comparison of fusion and reverse distances (in Å) in 47 fusion events from full-length proteins in which fusion occurs in only a single gene order; black bars represent the medians, and boxes and whiskers indicate the distribution quartiles.

See also Table S4.

are selected. Since the vast majority of hypothetical fusion events would modify existing assembly pathways, this helps to rationalize why most protein interactions are not predicted by fusion-based methods (e.g., only 3.7% of the nonredundant subunit pairs in our data set are associated with evolutionary fusion events). In addition, we demonstrated further selective pressure upon fusion related to assembly optimization and the requirement for covalent linkage of termini.

These findings provide a more detailed, structural understanding of fusion that should allow one to better interpret and utilize fusion-based predictions. Furthermore, fusion-based strategies have been gaining prominence in the field of protein engineering (Padilla et al., 2001; Sinclair et al., 2011; Lai et al., 2012). Our insights can also potentially guide future protein engineering approaches: if covalent fusion of subunits is desired in order to stabilize a complex, success is most likely to be achieved with engineered fusions that conserve existing assembly pathways and in which the gene order is chosen to best match the existing quaternary structure.

This work also reveals an evolutionary connection between protein and genome structure. In 13% of the cases we examined, fusion occurred in both orders (i.e., AB and BA), in similarity to previous work showing that the vast majority ($\sim$92%) of domain pairs occur in only a single order (Apic et al., 2001). It has been suggested that the order of domain combinations in multidomain proteins is due primarily to historical chance, as domain pairs with the same structure and function can occur in both orders given the presence of a long interdomain linker (Bashton and Chothia, 2002; Vogel et al., 2004). Thus, multidomain proteins are highly versatile and a short interterminal fusion distance is not a strict requirement. However, our results suggest that the formation of a long linker (as required to preserve the quaternary interaction) can be a limiting factor, because we observe a strong preference for fusions in the order corresponding to the shorter interterminal distance. Therefore, our work implies that, rather than being an evolutionary artifact, the order in which genes fuse can be directly related to the structural features of the proteins they encode, thus demonstrating a simple way in which protein structure can influence genomic organization.

Finally, our results highlight a fascinating connection between evolutionary processes, which act over millions of years, and assembly, which occurs on the order of seconds. Although the assembly pathways of homomeric complexes were previously found to reflect their evolutionary histories (Levy et al., 2008), here we observed an opposite phenomenon in which the evolutionary process of gene fusion mimics heteromer assembly in order to conserve the existing assembly pathway.

EXPERIMENTAL PROCEDURES

Structural Data Sets

We started with the full set of heteromeric biological units from protein crystal structures in the RCSB Protein Data Bank (Berman et al., 2000). We filtered heteromers formed by polypeptide cleavage by identifying different chains with the same external database reference identifier (*db_id*, which generally corresponds to the UniProt sequence) but with a sequence identity of <90%. Only subunits with at least 50 residues were considered. Protein complexes containing nucleic acids were ignored because we have no way of reliably predicting (dis)assembly for these cases.

We filtered subunit pairs from the protein complexes for redundancy, first by grouping them by their SUPERFAMILY domain assignments (Gough et al., 2001) and then by calculating the sequence identities between all pairs in each group. If both subunits from a pair had >70% sequence identity to another pair, only the pair from the higher-resolution crystal structure was kept. After the sequence redundancy filtering was completed, we had a total of 2,544 nonredundant heteromeric subunit pairs. All subunit pairs used in this study, along with their various relevant properties, are provided in Table S1.

For each complex, we calculated the size of the interfaces between all pairs of subunits using AREAIMOL (Collaborative Computational Project, Number 4, 1994). In complexes containing more than one copy of each subunit, there can be more than one interface for a given pair of subunit types (e.g., the two different α-β interfaces in 2F9Y; see Figure 2A). Therefore, in compiling our nonredundant set of subunit pairs, we only considered the largest interface

for a given pair of subunit types from each complex (e.g., only the largest α-β interface in 2F9Y). Pairs of subunits were considered to be directly interacting if they buried >200 Å^2 of intermolecular interface area.

For each pair of subunits, we searched the STRING v9.0 database (Szklarczyk et al., 2011) for evidence of fusion occurring between the genes encoding those subunits. This was defined as two proteins with a STRING fusion evidence score > 0.3, and each having >50% sequence identity to one of the interacting subunits. Note that STRING uses stringent criteria for identifying gene fusion events based upon orthology to nonfused genes, thus avoiding the requirement to filter putative fusion events involving promiscuous domains, as arises with homology-based approaches (Marcotte et al., 1999a). The significance of all of our results remains robust to the choice of STRNG evidence score (see Extended Experimental Procedures). Subunit pairs were thus divided into fusion pairs (having evidence of fusion between them) and nonfusion pairs (no evidence of fusion). For some complexes, multiple distinct fusion pairs were identified. In a few of these cases, STRING also identified indirect fusions. For example, in *K. aerogenes* urease, γ fuses with β and β fuses with α, but STRING also identified a γ-α fusion due to the indirect linkage via β. We manually identified these indirect fusion pairs in STRING and moved them to the nonfusion set. In total, 94 (3.7%) of the nonredundant heteromeric subunit pairs were associated with evolutionary gene fusion events.

In this study, we identified gene fusion events as cases in which two separate genes became joined. However, it is possible that some of these cases resulted from gene fission events (i.e., a prefusion complex was really a post-fission complex). Although this could potentially have some implications for our results, there is strong evidence that gene fusion is both the most dominant mechanism behind the evolution of multidomain proteins (Pasek et al., 2006; Buljan et al., 2010) and is much more common than gene fission (Kummerfeld and Teichmann, 2005; Fong et al., 2007). This suggests that any contribution of fission to our data set must be minimal and therefore unable to account for the strong trends we observed.

High-Throughput Protein Interaction Data

Just as we identified the subunit pairs from crystal structures, we compiled analogous data sets from high-throughput protein-protein interaction data. Instead of using crystal structures, we identified interacting pairs of proteins as those with evidence of interaction in the STRING database (experimental evidence score > 0.3). We could then directly split these interacting pairs into fusion and nonfusion pairs using the STRING fusion evidence score.

nESI-MS Experiments

The complexes were kindly donated as follows: *Salmonella typhimurium* tryptophan synthase (Protein Data Bank [PDB] ID: 1WBJ; I. Schlichting, Max Planck Institute for Medical Research, Heidelberg); *E. coli* acetyl coA carboxylase carboxyltransferase (PDB ID: 2F9Y; G. Waldrop, Louisiana State University); *E. coli* carbamoyl phosphate synthetase (PDB ID: 1BXR; F. Raushel, Texas A&M University); and *K. aerogenes* and *Helicobacter mustelae* ureases (PDB ID: 1KRA and 3QGA, respectively; R. Hausinger, Michigan State University). Complexes were buffer exchanged from their purification buffers to ammonium acetate at near-neutral pH, and further diluted with ammonium acetate to give solutions containing 0.5–8 μM complex in 60–250 mM ammonium acetate. Concentrations were adjusted for each complex to yield spectra of the intact complex, and all subsequent solution disruption experiments used the same complex and ammonium acetate concentration as a starting point. Solution disruption was carried out by addition of one or more of the following: methanol, ethanol, 2-propanol, acetonitrile, dimethyl sulfoxide, acetic acid, ammonia solution, ammonium acetate, and water.

Mass spectra were acquired using QToF2 or Synapt HDMS G2 (Waters, Manchester, UK) instruments, modified for high m/z operation (Sobott et al., 2002), in positive ion nESI mode. Samples were introduced using borosilicate capillaries drawn to a fine tip and gold coated in-house. For each complex, we explored a range of voltage and pressure conditions in order to detect subcomplexes between the m/z values of the intact complex and free subunits (Hernández and Robinson, 2007). Subcomplex identities were confirmed by MS/MS spectra.

A high concentration (4–7 μM) of the complex was used to investigate the extent of reassembly after the addition of acetic acid, ammonia solution, or organic solvents. Aliquots of the concentrated disassembly solution were diluted to the same complex concentration with either the buffer/solvent mix or ammonium acetate alone. A control solution was also prepared from the complex in ammonium acetate buffer to obtain solution conditions identical to those of the reassembly solution. Spectra from the three solutions were acquired using identical MS conditions.

In Silico (Dis)assembly

(Dis)assembly pathways were predicted for all heteromeric complexes with more than three total subunits. We used a simple model based upon interface size in which a complex was iteratively dissociated so that each step required the disruption of the smallest total interface area.

For each pair of subunits associated with a fusion event, a heteromeric pair of subunits from the same complex was randomly selected, giving 36 fusion pairs and 36 randomly selected pairs. The mean value of the property of interest for the fusion pairs (e.g., conservation of [dis]assembly or reduction of interfaces upon fusion) was compared with the mean value from the randomly selected pairs. The procedure was repeated 10^6 times, allowing the p value to be calculated as the frequency with which the random pairs had a mean value less than or equal to that of the fusion pairs (i.e., the chance that the mean value could be observed if fusions occurred randomly in the same complexes). A Perl script for performing this analysis is provided in the Extended Experimental Procedures.

We also performed a similar comparison of shared interaction partners from the protein-protein interaction data. Instead of comparing fusion pairs with random pairs from the same complex, we compared them with random pairs from the same interaction network. For example, given a fusion pair, A and B, we also considered all of the interactions involving A or B, as well as the interactions between proteins that both interacted with A or B. To calculate the p values, we repeated the process 10^4 times, and determined the likelihood that the observed value could have been seen by chance. A Perl script for performing this calculation is provided in the Extended Experimental Procedures. This analysis was performed for all of the STRING “core” species (Table S3).

Terminal Distance Calculations

The distance between the N and C termini of different chains was calculated as the distance between the Cα atoms of their terminal residues. Since the N and C termini present in crystal structures may not represent the actual biologically relevant termini, for this analysis we used only full-length proteins, and filtered out fusion events in which any of the termini were missing (e.g., due to disorder or the expression construct). We did this by identifying subunits in which the 20 N- or C-terminal residues from the full-length protein were missing. We identified the sequences of the full-length proteins by performing a *blastp* (Altschul et al., 1997) search against all proteins in the STRING database and selecting the sequence with the lowest E value. We determined the order of gene fusion (i.e., AB or BA) by manually noting the order in which the genes are fused in the STRING web interface. All of the fusion and reverse distances are provided in Table S4.

ACCESSION NUMBERS

The protein interactions from this publication have been submitted to the IMEx consortium (http://www.imexconsortium.org) through IntAct (PMID: 19850723) and assigned the identifier IM-18676.

SUPPLEMENTAL INFORMATION

Supplemental Information includes Extended Experimental Procedures, three figures, and four tables and can be found with this article online at http://dx.doi.org/10.1016/j.cell.2013.02.044.

ACKNOWLEDGMENTS

We thank I. Schlichting (Max Planck Institute for Medical Research, Heidelberg), G. Waldrop (Louisiana State University), F. Raushel (Texas A&M University), and R. Hausinger (Michigan State University) for providing protein

complex samples. We acknowledge E. Boeri-Erba and K. Wright for assistance with the MS experiments, and M. Babu and E. Natan for comments on the manuscript. J.A.M., T.P., and S.A.T. were supported by the Medical Research Council (MRC file reference number U105161047). J.A.M. was supported by a long-term fellowship from the Human Frontier Science Program Organization. H.H. was supported by an MRC Programme Grant. S.E.A. was supported by the Royal Society. C.V.R. was supported by an ERC Advanced Grant and a Royal Society Professorship.

Received: December 18, 2012
Revised: February 5, 2013
Accepted: February 21, 2013
Published: April 11, 2013

REFERENCES

Altschul, S.F., Madden, T.L., Schäffer, A.A., Zhang, J., Zhang, Z., Miller, W., and Lipman, D.J. (1997). Gapped BLAST and PSI-BLAST: a new generation of protein database search programs. Nucleic Acids Res. *25*, 3389–3402.

Apic, G., Gough, J., and Teichmann, S.A. (2001). Domain combinations in archaeal, eubacterial and eukaryotic proteomes. J. Mol. Biol. *310*, 311–325.

Baldick, C.J., Jr., Cassetti, M.C., Harris, N., and Moss, B. (1994). Ordered assembly of a functional preinitiation transcription complex, containing vaccinia virus early transcription factor and RNA polymerase, on an immobilized template. J. Virol. *68*, 6052–6056.

Bashton, M., and Chothia, C. (2002). The geometry of domain combination in proteins. J. Mol. Biol. *315*, 927–939.

Berman, H.M., Westbrook, J., Feng, Z., Gilliland, G., Bhat, T.N., Weissig, H., Shindyalov, I.N., and Bourne, P.E. (2000). The Protein Data Bank. Nucleic Acids Res. *28*, 235–242.

Björklund, A.K., Ekman, D., Light, S., Frey-Skött, J., and Elofsson, A. (2005). Domain rearrangements in protein evolution. J. Mol. Biol. *353*, 911–923.

Brooijmans, N., Sharp, K.A., and Kuntz, I.D. (2002). Stability of macromolecular complexes. Proteins *48*, 645–653.

Buljan, M., Frankish, A., and Bateman, A. (2010). Quantifying the mechanisms of domain gain in animal proteins. Genome Biol. *11*, R74.

Collaborative Computational Project, Number 4. (1994). The CCP4 suite: programs for protein crystallography. Acta Crystallogr. D Biol. Crystallogr. *50*, 760–763.

Dobson, C.M. (2003). Protein folding and misfolding. Nature *426*, 884–890.

Durand, A., and Merrick, M. (2006). In vitro analysis of the Escherichia coli AmtB-GlnK complex reveals a stoichiometric interaction and sensitivity to ATP and 2-oxoglutarate. J. Biol. Chem. *281*, 29558–29567.

Ellis, R.J. (2007). Protein misassembly: macromolecular crowding and molecular chaperones. Adv. Exp. Med. Biol. *594*, 1–13.

Enright, A.J., and Ouzounis, C.A. (2001). Functional associations of proteins in entire genomes by means of exhaustive detection of gene fusions. Genome Biol. *2*, RESEARCH0034.

Enright, A.J., Iliopoulos, I., Kyrpides, N.C., and Ouzounis, C.A. (1999). Protein interaction maps for complete genomes based on gene fusion events. Nature *402*, 86–90.

Evans, D.R., Pastra-Landis, S.C., and Lipscomb, W.N. (1974). An intermediate complex in the dissociation of aspartate transcarbamylase. Proc. Natl. Acad. Sci. USA *71*, 1351–1355.

Fong, J.H., Geer, L.Y., Panchenko, A.R., and Bryant, S.H. (2007). Modeling the evolution of protein domain architectures using maximum parsimony. J. Mol. Biol. *366*, 307–315.

Friedman, F.K., and Beychok, S. (1979). Probes of subunit assembly and reconstitution pathways in multisubunit proteins. Annu. Rev. Biochem. *48*, 217–250.

Gallastegui, N., and Groll, M. (2010). The 26S proteasome: assembly and function of a destructive machine. Trends Biochem. Sci. *35*, 634–642.

Gough, J., Karplus, K., Hughey, R., and Chothia, C. (2001). Assignment of homology to genome sequences using a library of hidden Markov models that represent all proteins of known structure. J. Mol. Biol. *313*, 903–919.

Hernández, H., and Robinson, C.V. (2007). Determining the stoichiometry and interactions of macromolecular assemblies from mass spectrometry. Nat. Protoc. *2*, 715–726.

Jabri, E., and Karplus, P.A. (1996). Structures of the Klebsiella aerogenes urease apoenzyme and two active-site mutants. Biochemistry *35*, 10616–10626.

Janin, J., Rodier, F., Chakrabarti, P., and Bahadur, R.P. (2007). Macromolecular recognition in the Protein Data Bank. Acta Crystallogr. D Biol. Crystallogr. *63*, 1–8.

Kamburov, A., Goldovsky, L., Freilich, S., Kapazoglou, A., Kunin, V., Enright, A.J., Tsaftaris, A., and Ouzounis, C.A. (2007). Denoising inferred functional association networks obtained by gene fusion analysis. BMC Genomics *8*, 460.

Kim, P.M., Lu, L.J., Xia, Y., and Gerstein, M.B. (2006a). Relating three-dimensional structures to protein networks provides evolutionary insights. Science *314*, 1938–1941.

Kim, W.K., Henschel, A., Winter, C., and Schroeder, M. (2006b). The many faces of protein-protein interactions: A compendium of interface geometry. PLoS Comput. Biol. *2*, e124.

Kühner, S., van Noort, V., Betts, M.J., Leo-Macias, A., Batisse, C., Rode, M., Yamada, T., Maier, T., Bader, S., Beltran-Alvarez, P., et al. (2009). Proteome organization in a genome-reduced bacterium. Science *326*, 1235–1240.

Kummerfeld, S.K., and Teichmann, S.A. (2005). Relative rates of gene fusion and fission in multi-domain proteins. Trends Genet. *21*, 25–30.

Lai, Y.-T., Cascio, D., and Yeates, T.O. (2012). Structure of a 16-nm cage designed by using protein oligomers. Science *336*, 1129.

Levinthal, C. (1969). How to fold graciously. In Mossbauer Spectroscopy in Biological Systems: Proceedings of a Meeting Held at Allerton House, Monticello, Illinois, J.T.P. DeBrunner and E. Munck, eds. (Champaign, IL: University of Illinois Press), pp. 22–24.

Levy, E.D., Boeri Erba, E., Robinson, C.V., and Teichmann, S.A. (2008). Assembly reflects evolution of protein complexes. Nature *453*, 1262–1265.

Lindorff-Larsen, K., Piana, S., Dror, R.O., and Shaw, D.E. (2011). How fast-folding proteins fold. Science *334*, 517–520.

Marcotte, C.J.V., and Marcotte, E.M. (2002). Predicting functional linkages from gene fusions with confidence. Appl. Bioinformatics *1*, 93–100.

Marcotte, E.M., Pellegrini, M., Ng, H.L., Rice, D.W., Yeates, T.O., and Eisenberg, D. (1999a). Detecting protein function and protein-protein interactions from genome sequences. Science *285*, 751–753.

Marcotte, E.M., Pellegrini, M., Thompson, M.J., Yeates, T.O., and Eisenberg, D. (1999b). A combined algorithm for genome-wide prediction of protein function. Nature *402*, 83–86.

Marsh, J.A., and Teichmann, S.A. (2011). Relative solvent accessible surface area predicts protein conformational changes upon binding. Structure *19*, 859–867.

Marsh, J.A., Teichmann, S.A., and Forman-Kay, J.D. (2012). Probing the diverse landscape of protein flexibility and binding. Curr. Opin. Struct. Biol. *22*, 643–650.

Nooren, I.M.A., and Thornton, J.M. (2003). Diversity of protein-protein interactions. EMBO J. *22*, 3486–3492.

Padilla, J.E., Colovos, C., and Yeates, T.O. (2001). Nanohedra: using symmetry to design self assembling protein cages, layers, crystals, and filaments. Proc. Natl. Acad. Sci. USA *98*, 2217–2221.

Pasek, S., Risler, J.-L., and Brézellec, P. (2006). Gene fusion/fission is a major contributor to evolution of multi-domain bacterial proteins. Bioinformatics *22*, 1418–1423.

Payne, M.S., Wu, S., Fallon, R.D., Tudor, G., Stieglitz, B., Turner, I.M., Jr., and Nelson, M.J. (1997). A stereoselective cobalt-containing nitrile hydratase. Biochemistry *36*, 5447–5454.

Perica, T., Marsh, J.A., Sousa, F.L., Natan, E., Colwell, L.J., Ahnert, S.E., and Teichmann, S.A. (2012). The emergence of protein complexes: quaternary

structure, dynamics and allostery. Colworth Medal Lecture. Biochem. Soc. Trans. *40*, 475–491.

Poulsen, C., Bongaerts, R.J., and Verpoorte, R. (1993). Purification and characterization of anthranilate synthase from Catharanthus roseus. Eur. J. Biochem. *212*, 431–440.

Raker, V.A., Plessel, G., and Lührmann, R. (1996). The snRNP core assembly pathway: identification of stable core protein heteromeric complexes and an snRNP subcore particle in vitro. EMBO J. *15*, 2256–2269.

Reid, A.J., Ranea, J.A.G., Clegg, A.B., and Orengo, C.A. (2010). CODA: accurate detection of functional associations between proteins in eukaryotic genomes using domain fusion. PLoS ONE *5*, e10908.

Shoemaker, B.A., and Panchenko, A.R. (2007). Deciphering protein-protein interactions. Part I. Experimental techniques and databases. PLoS Comput. Biol. *3*, e42.

Sinclair, J.C., Davies, K.M., Vénien-Bryan, C., and Noble, M.E.M. (2011). Generation of protein lattices by fusing proteins with matching rotational symmetry. Nat. Nanotechnol. *6*, 558–562.

Sobott, F., Hernández, H., McCammon, M.G., Tito, M.A., and Robinson, C.V. (2002). A tandem mass spectrometer for improved transmission and analysis of large macromolecular assemblies. Anal. Chem. *74*, 1402–1407.

Szklarczyk, D., Franceschini, A., Kuhn, M., Simonovic, M., Roth, A., Minguez, P., Doerks, T., Stark, M., Muller, J., Bork, P., et al. (2011). The STRING database in 2011: functional interaction networks of proteins, globally integrated and scored. Nucleic Acids Res. *39*(Database issue), D561–D568.

Vogel, C., Bashton, M., Kerrison, N.D., Chothia, C., and Teichmann, S.A. (2004). Structure, function and evolution of multidomain proteins. Curr. Opin. Struct. Biol. *14*, 208–216.

Yanai, I., Derti, A., and DeLisi, C. (2001). Genes linked by fusion events are generally of the same functional category: a systematic analysis of 30 microbial genomes. Proc. Natl. Acad. Sci. USA *98*, 7940–7945.

EXTENDED EXPERIMENTAL PROCEDURES

(Dis)Assembly Pathways from Mass Spectrometry Experiments

Nano-electrospray mass spectrometry (nESI-MS) experiments were performed on each complex under native and destabilizing conditions. The experimentally determined in vitro (dis)assembly pathways are shown in Figure 2A. The pathways identified for each complex are discussed in detail here.

Carbamoyl phosphate synthetase from *E. coli* (PDB ID: 1BXR) contains four copies of each of the two distinct subunits, α and β (Thoden et al., 1999). (Dis)assembly occurs via two stages, involving the breaking of the two different types of α-α interfaces. Notably, this pathway is consistent with the different oligomeric states that have been observed for this complex in the presence of different small molecules and at different concentrations (Kim and Raushel, 2001). We also see evidence for smaller populations of both intermediate subcomplexes under native conditions, suggesting that an equilibrium (dis)assembly process is occurring. This is interesting when considering that PISA (Krissinel and Henrick, 2007) predicts $\alpha\beta$ to be the only stable complex in solution. Since the $\alpha\beta$ pair stays associated, the gene fusion event therefore conserves the (dis)assembly pathway. Note however that there is some ambiguity regarding the identity of the intermediate $\alpha_2\beta_2$ subcomplex because there are two ways (*i.e.* two distinct α-α interfaces) in which it could be formed. While our experiments were unable to distinguish between the two $\alpha_2\beta_2$ forms, mutational evidence suggests that this state would retain the larger α-α interface of the "allosteric domain" (Kim and Raushel, 2001), which is consistent with the (dis)assembly prediction. Thus, although there can be a very minor population of $\alpha_3\beta_3$ formed under some conditions that is not predicted (Table S2), the interface-size model predicts the (dis)assembly pathway almost perfectly.

Tryptophan synthase from *Salmonella typhimurium* (PDB ID: 1WBJ) has two distinct subunits, α and β, with the full complex containing two copies of each (Kulik et al., 2005). In our experiments, (dis)assembly of this complex occurs via the breaking of the α-β interfaces to give $\alpha\beta_2$, β_2 and α monomers. Thus, fusion between α and β would change the (dis)assembly pathway and (dis)assembly would not be conserved. However, since the fusion event does not involve the largest interface, the experimentally observed (dis) assembly pathway is in full agreement with prediction.

Acetyl-CoA carboxylase carboxyltransferase from *E. coli* (PDB ID: 2F9Y) contains two of each α and β subunits (Bilder et al., 2006). Our experiments demonstrate that this complex disassembles via an $\alpha\beta$ subcomplex, with no intermediate states (*i.e.* $\alpha\beta_2$ or $\alpha_2\beta$) observed. Interestingly, in contrast to the other experimentally characterized prefusion complexes where high subcomplex populations are observed, our experiments demonstrate that the overall $\alpha\beta$ population is fairly low, and that most subunits dissociate to α and β monomers under the conditions examined. Thus the $\alpha\beta$ dimer appears to be of fairly low overall stability. Similar to carbamoyl phosphate synthetase above, there are two possibilities for the $\alpha\beta$ subcomplex. Fortunately, we were able to use ion mobility (IM)-MS to distinguish between these, by comparing the measured collision cross section (CCS) to predicted values for the two subcomplexes calculated from the crystal structure (Figure S1). This shows that the observed $\alpha\beta$ dimer corresponds to the larger interface, and the (dis)assembly pathway is the same as predicted. In addition the α and β subunits stay together (as opposed to the alternate possibilities involving loss of individual α or β subunits) and the α-β fusion event would conserve (dis)assembly.

Klebsiella aerogenes urease (PDB ID: 1KRA) has three distinct subunits, α, β and γ, which are each repeated three times in the full complex (Jabri and Karplus, 1996). The first steps of (dis)assembly occur exactly as predicted, with the sequential loss of two of the three β subunits. However, from this point, there is some deviation from prediction and two distinct pathways are observed. In one, the third β subunit is lost (in accordance with prediction), while in the other an α subunit is lost (not predicted). Finally, one or both of these subcomplexes split into a γ_3 trimer and an α_2 dimer. Despite the apparent complexity of this pathway, we can clearly see that neither of the interfaces that fuse (α-β or β-γ) is maintained upon (dis)assembly, since the β subunit is the first to dissociate. Thus the (dis)assembly pathway would not be conserved upon fusion.

Finally, we probed the (dis)assembly of the urease complex from *Helicobacter mustelae* (PDB ID: 3QGA) (Carter et al., 2011). MS confirmed that this complex contains 12 copies of each of the α and β subunits. Thus its overall tetrahedral symmetry is equivalent to four repeats of the full *K. aerogenes* urease, which has C_3 symmetry. The first three steps of (dis)assembly involve dissociation of individual $\alpha_3\beta_3$ subcomplexes, exactly as predicted, and consistent with a previous study of *H. pylori* urease (Pinkse et al., 2003). Following this, there are two separate pathways observed. In one, an α subunit first dissociates, which is contrary to prediction, but the rest of (dis)assembly proceeds as predicted, leading to an $\alpha\beta$ dimer. In the other pathway, a β_2 dimer is first lost, as opposed to the sequential loss of two β subunits that is predicted. From here (dis)assembly also proceeds as predicted to an $\alpha\beta$ dimer, while an alternate, unpredicted pathway leads to α_3 and α_2. There are two $\alpha\beta$ subcomplexes that could potentially form due to the two different α-β interfaces. Although we have assumed that the observed $\alpha\beta$ subcomplex corresponds to the larger interface, we cannot say for certain. Furthermore, despite the fact that one $\alpha\beta$ pair stays together, there is still some α-β dissociation (e.g., $\alpha_3\beta_3 \rightarrow \alpha_2\beta_3$). Thus fusion would partially modify (dis)assembly, and (dis)assembly would not be conserved.

Two arrows in the *H. mustelae* urease (dis)assembly pathway in Figure 2A have been colored blue. This is because the subcomplexes seen on either side were not seen under similar solution conditions. The $\alpha_3\beta$ and $a_2\beta$ subcomplexes were only observed under acidic conditions while $\alpha_2\beta_2$ and $\alpha\beta$ were observed under less acidic ($\alpha_2\beta_2$) or acetonitrile/basic conditions (both subcomplexes; see Table S2). The pathway presented here represents the simplest way of linking all of the subcomplexes observed under different conditions. However, it is possible that the $\alpha_2\beta_2$ subcomplex may directly dissociate to an $\alpha\beta$ dimer under the conditions tested.

Literature-Identified (Dis)Assembly Pathways

In addition to the experimentally characterized complexes, we identified four examples of prefusion complexes for which (dis)assembly pathways could be determined from previously published studies. These pathways are shown in Figure 2B.

The (dis)assembly pathway for cobalt-containing nitrile hydratase from *Pseudomonas putida* (PDB ID: 3QXE) (Brodkin et al., 2011) was determined from gel filtration experiments which showed that the protein exists in both $\alpha\beta$ and $(\alpha\beta)_2$ forms (Payne et al., 1997). The biological unit for this complex consists of an $\alpha\beta$ form, while the asymmetric unit contains $(\alpha\beta)_4$. Therefore we used the $(\alpha\beta)_2$ form of the complex predicted from PISA (Krissinel and Henrick, 2007). The (dis)assembly pathway for this complex therefore goes from $(\alpha\beta)_2$ to $\alpha\beta$ which is consistent with the interface-size prediction and shows that (dis)assembly would be conserved upon fusion.

The (dis)assembly pathways for the AmtB-GlnK complex from *E. coli* (PDB ID: 2NS1) (Gruswitz et al., 2007) was also determined from gel filtration experiments (Durand and Merrick, 2006). Under native conditions, the complex ran at a size consistent with the A_3G_3 form of the crystal structure. However, under high salt conditions, the complex dissociated into A_3 and G_3 forms. This is exactly as predicted from the interface sizes of the full complex, but shows that fusion would not conserve (dis)assembly as the A and G subunits dissociate from each other.

The (dis)assembly pathway for aspartate transcarbamoylase from *E. coli* (PDB ID: 1D09) (Jin et al., 1999), which contains 6 catalytic (C) and 6 regulatory (R) subunits was determined through the use of mercurials, which react with the sulfhydryl groups of cysteine residues and can disrupt interfaces (Evans et al., 1974). Partial dissociation with mercurials leads to an intermediate C_6R_4 form of the complex, suggesting that (dis)assembly occurs via loss of R_2 regulatory dimers, while complete dissociation leads to the C_3 catalytic trimer. While not all steps in the full predicted (dis)assembly pathway have been observed, the observed subcomplexes are in full agreement with prediction and demonstrate that the (dis)assembly pathway of this complex would not be conserved upon fusion.

The (dis)assembly pathway of anthanilate synthase from *Catharanthus roseus* was determined from gel filtration experiments which showed that the complex elutes as a $(\alpha\beta)_2$ tetramer under native conditions but as an $\alpha\beta$ dimer at higher salt concentrations (Poulsen et al., 1993). Although no crystal structure has been published for the enzyme from this organism, there are published crystal structures of homologs with the same $(\alpha\beta)_2$ topology. Therefore, we used the most closely related structure (PDB ID: 1I7Q) (Spraggon et al., 2001). The experimentally observed (dis)assembly pathway agrees with prediction and demonstrates that fusion between the α and β subunits would conserve (dis)assembly.

Reassembly of Heteromeric Complexes

The experiments performed in this study involve the in vitro disassembly of protein complexes. Starting from the full complex under native conditions, increasingly destabilizing solution conditions are applied and the dissociated subcomplexes are observed by MS. This is analogous to protein unfolding experiments, in which the loss of secondary and tertiary structure can be traced under increasing concentrations of denaturant. However, just as chemical denaturation may be different than folding under native conditions, the assembly of a protein complex could conceivably follow a different pathway than observed for in vitro disassembly.

One way of gaining confidence in the correspondence between assembly and disassembly is by performing reassembly experiments in which the dissociated complex is returned to native solution conditions. If the full complex can be reassembled from the dissociated state, and no alternate, off-pathway subcomplexes are observed, then the disassembly pathway is essentially confirmed to be the reverse of assembly. Previously, reassembly experiments were used to demonstrate this in homomeric complexes (Levy et al., 2008). Here, we have adopted a similar approach and performed reassembly experiments on the heteromeric prefusion complexes. For each complex, mass spectra are provided under destabilizing, reassembled and native conditions (Figure S2). Reassembly is assessed by comparing the reassembled complex to the native complex.

For carbamoyl phosphate synthetase and tryptophan synthase, the reassembled complexes match the native complexes very closely, indicating that full reassembly has occurred.

For *K. aerogenes* urease, the full complex is also reformed upon reassembly. However, we do note that the overall yield of reassembled complex appears to be lower, as evidenced by the relatively weak signal to noise in this spectrum. Nevertheless, the full complex is the only species observed.

For *H. mustelae* urease, good reassembly of the full complex is achieved. Note however that the reassembled spectrum provided here was only obtained 24 hr after the start of reassembly. For all other complexes, the full reassembled spectra were obtained within one hour. Spectra taken within one hour for this complex showed that reassembly has hardly occurred, indicating that reassembly is a much slower kinetic process for this complex than for the others.

Reassembly experiments were not performed for acetyl-CoA carboxylase carboxyltransferase because all available protein sample was used in the disassembly experiments. However, given the relatively simple topology of this complex and its observed disassembly pathway ($\alpha_2\beta_2 \rightarrow \alpha\beta$), it is difficult to imagine any alternate reassembly pathway. Due to the results obtained here, as well as the previous results for homomers (Levy et al., 2008), we have strong confidence that the assembly pathway for this complex is also likely to be the reverse of disassembly.

Further confidence in the in vitro disassembly pathways identified here is obtained by noting that we generally see the same subcomplexes forming under a range of solution conditions (Table S2). Thus the pathways observed are not strongly dependent on the choice of destabilizing agent. The exception to this is the *Helicobacter musteale* urease, for which some differences were observed, as noted in earlier.

Fusion in Obligate Complexes

Large interfaces have been noted as characteristic of obligate complexes, in which the subunits are permanently associated within the cell (Nooren and Thornton, 2003). These can be contrasted with transient complexes, which have smaller interfaces and for which the free subunits can often be observed in isolation. Since experimental (dis)assembly pathways appear to be driven primarily by interface size, we wondered whether fusion may preferentially occur in obligate complexes that form large intermolecular interfaces.

In the main text, we primarily compared evolutionarily observed fusion events to a null model in which fusion events occurred randomly within the same complexes. Here, instead, we compare pairs of subunits known to undergo fusion (“fusion pairs”) to pairs of subunits from heteromeric complexes with no evolutionary evidence for fusion (“nonfusion pairs”).

First, we compared the interface sizes of fusion pairs versus nonfusion pairs. The mean interface size of the fusion pairs (3612 Å^2) is much larger than observed for the nonfusion pairs (1899 Å^2, $p < 2.2 \times 10^{-16}$, Wilcoxon rank-sum test), demonstrating that fusion preferentially occurs between subunits forming large interfaces (Figure S3A). Similarly, using the random model, we see a mean interface size of 4,167 Å^2 associated with fusion events versus 2,302 Å^2 for random fusions within the same complexes ($p < 1 \times 10^{-6}$).

Next, we searched for complexes with structural evidence for being transient (*i.e.* a monomeric crystal structure is available for at least one subunit). These were defined as protein complexes with subunits having > 70% sequence identity to a monomeric crystal structure. Any pair of subunits for which there is a monomeric structure corresponding to at least one of them was considered to be transient. Figure S3B shows that only 19.1% of fusion pairs involve transient subunits, compared to 40.0% of nonfusion pairs ($p = 3 \times 10^{-5}$, Fisher’s exact test). Thus we see that subunits that undergo fusion are rarely observed in isolation, whereas this is quite common for protein complexes in general, suggesting an evolutionary preference for gene fusion events in obligate complexes.

An additional way of identifying transient versus obligate complexes is by using mRNA coexpression data. If two genes show correlated coexpression, then they are more likely to be obligate since they will usually be expressed together at all times (Jansen et al., 2002). Therefore, we identified coexpressed pairs of subunits using the coexpression score (>0.3) from STRING, similar to how subunit pairs associated with fusion events were identified. In Figure S3C, we observe a substantial increase in the fraction being coexpressed (*i.e.* from likely obligate complexes) for fusion pairs (48.9%) versus nonfusion pairs (21.8%, $p = 2 \times 10^{-8}$, Fisher’s exact test).

We also performed a similar analysis using nonstructural protein interaction data. We considered all pairs of *Escherichia coli* proteins with experimental evidence for interaction in the STRING database. Of those pairs that undergo fusion, 66.1% are coexpressed, compared to only 16.9% of nonfusion interacting pairs ($p < 2.2 \times 10^{-16}$, Fisher’s exact test) (Figure S3D). Similar trends are seen in *Saccharomyces cerevisiae* (56.3% of fusion events versus 8.6% of nonfusion pairs, $p = 2 \times 10^{-6}$) and *Homo sapiens* (70.6% versus 11.1%, $p = 5 \times 10^{-13}$). Note that while a previous study showed higher coexpression between *S. cerevisiae* genes that fuse compared to random (not necessarily interacting) pairs (Marcotte et al., 1999b), this analysis only considers coexpression between interacting pairs. Thus, assuming that coexpression can be used a reasonable proxy for the obligateness of a complex, these results strongly support the idea fusion has a much greater propensity to occur between subunits in obligate complexes compared to transient complexes.

Since obligate complexes tend to have large interfaces, it might be suggested that the observed tendency for (dis)assembly conservation could reflect possible selection for fusion events involving obligate complexes. However, there is a strong argument against this in that we demonstrated a significant tendency for evolutionary gene fusion events to conserve (dis)assembly, *compared to random fusions occurring within the same complexes* (Figure 3C). Thus, the only way the observed (dis)assembly conservation could be due to a preference for fusion in obligate complexes is if some of the prefusion complexes in our data set have both obligate and transient subunits.

To show that fusion does not preferentially involve the more obligate subunits of prefusion complexes, we compared the transientness and obligateness of subunits associated with fusion events to what we expect for random fusions within the same complex. First, we observe that 8.3% of fusion events in complexes with > 2 distinct subunits involve at least one transient subunit (*i.e.* a monomeric crystal structure exists), compared to 9.1% expected by chance, which is far from significant ($p = 0.6$). Furthermore, we observe that the percentage of fusion events that involve subunits with correlated coexpression (33.3%) is in fact lower than expected for random fusions within the same complexes (36.0%), although this difference is also not significant ($p = 0.4$). Therefore, these results argue against any evolutionary preference for fusions involving more obligately associated subunits at the *intracomplex level*, instead suggesting that selection for assembly conservation plays a more important role.

To further demonstrate that the observed tendency for (dis)assembly conservation is independent of the preference for fusion in obligate complexes, we repeated our (dis)assembly conservation analyses considering only obligately associated subunits. First, we limited our data set to only pairs of subunits that showed evidence for correlated coexpression, which are thus likely to be obligately associated (Jansen et al., 2002). We observe evidence for fusion in 12/39 (30.8%) coexpressed subunit pairs in which a hypothetical fusion would conserve (dis)assembly, compared to only 20/401 (5.0%) in which fusion would modify (dis)assembly ($p = 3 \times 10^{-6}$, Fisher’s exact test). In addition, when using the random fusion model, we see that 45.5% of fusion events in complexes with > 2 distinct subunits conserve (dis)assembly, compared to only 19.6% expected by chance, which remains statistically significant ($p = 0.03$), despite the limited size of the data set ($n = 11$).

Next, we defined obligateness on the basis of the total amount of interface area buried by a subunit within a complex. When considering only subunits with > 4000 Å^2 total interface, we observe evidence for fusion in 20/128 (15.6%) of subunit pairs that would conserve (dis)assembly, compared to 33/801 (4.1%) in those that would modify (dis)assembly ($p = 6 \times 10^{-6}$, Fisher’s exact test). We observe (dis)assembly to be conserved in 38.7% of fusion events, compared to 14.8% expected by chance ($p = 2 \times 10^{-4}$,

n = 31). Similar statistically significant results were obtained by limiting the analyses to subunits with > 2,000 or 6,000 Å^2 total interface.

Together, these results demonstrate that the tendency for (dis)assembly conservation is strong, even when only obligately associated subunits are considered. Likewise, this newly identified preference for fusion events to occur in obligate complexes is independent of (dis)assembly conservation and likely represents an important functional constraint on gene fusion.

(Dis)Assembly Conservation Is Independent of the Tendency for Fusion between Closer Termini

In the main text, we demonstrated both a strong tendency for (dis)assembly pathways to be conserved upon fusion, and a significant preference for fusions involving closer termini. To show that these trends are not related, we considered only those fusion events in which the reverse distance is shorter than the fusion distance (*i.e.* fusion does not occur between the closer termini). With this limited data set, we still observed that 37.5% of fusion events in complexes with > 2 unique subunits conserved (dis)assembly, compared to only 17.7% expected by chance. This result remains significant (p = 0.04) in spite of the small size of the data set (n = 8). Thus, the tendency for (dis)assembly conservation appears to be independent of the tendency for fusion between closer termini.

Effects of Varying the STRING Evidence Threshold

For all results presented in this study, a STRING evidence threshold of 0.3 was used. The value of 0.3 was chosen as a level at which we could be fairly certain that most of the identified gene fusion events had actually occurred, as evidence of both the pre- and post-fused forms could be seen in multiple genomes for most cases. By lowering this threshold, we could increase the number of identified gene fusion events at the risk of including some false positives. To show that the significance of our results was not dependent on the STRING threshold, we repeated our analyses using the default low confidence (0.15) and high confidence (0.7) thresholds.

Using the low confidence threshold, we observe evidence for fusion in 25/201 (12.4%) of subunit pairs that would conserve (dis) assembly, compared to 53/1286 (4.1%) of those that would modify (dis)assembly ($p = 1 \times 10^{-5}$, Fisher's exact test). In addition, we observed that 35.0% of gene fusion events in complexes with > 2 unique subunits conserved (dis)assembly, compared to 14.7% expected by chance ($p = 2 \times 10^{-4}$, n = 40). Using the high confidence threshold, we observe evidence for fusion in 22/201 (10.9%) of subunit pairs that would conserve (dis)assembly, compared to 37/1286 (2.9%) in those that would modify (dis)assembly ($p = 2 \times 10^{-6}$, Fisher's exact test). In addition, we observed that 41.4% of gene fusion events in complexes with > 2 unique subunits conserved (dis)assembly, compared to 13.6% expected by chance ($p = 2 \times 10^{-5}$, n = 29).

Using the low confidence threshold, we observed that a mean of 2.66 interfaces were reduced upon fusion, compared to 2.18 expected by chance (p = 0.005). Using the high confidence threshold, we observed a mean of 3.21 interfaces reduced upon fusion, compared to 2.30 expected by chance ($p = 6 \times 10^{-6}$).

Using the low confidence threshold, we observed a mean of 11.1 versus 7.4% shared binding partners for *E. coli* ($p = 5 \times 10^{-4}$, n = 89), 9.0 versus 6.9% for yeast (p = 0.06, n = 40) and 15.5 versus 9.5% for human (p = 0.003, n = 28). Using the high confidence threshold, we observed a mean of 38.3 versus 27.8% shared binding partners for *E. coli* (p = 0.04, n = 15), 12.1 v. 8.1% for yeast (p = 0.13, n = 10) and 26.9 versus 18.1% for human (p = 0.12, n = 5). Thus we lose some statistical significance for this analysis using the high confidence threshold since it dramatically limits the number of fusion pairs with both fusion and experimental evidence scores > 0.7.

Using the low confidence threshold, we observed the fusion distance to be shorter than the reverse distance in 38/52 (73%) cases (p = 0.001, binomial test). With the high confidence threshold, the fusion distance was shorter than the reverse distance in 26/35 (74%) cases (p = 0.005, binomial test).

Significance of Results in Eukaryotes

A large majority of the subunit pairs associated with evolutionary gene fusion events came from prokaryotic species. Since fusion is a major mechanism for evolutionary domain gain (Björklund et al., 2005; Pasek et al., 2006; Buljan et al., 2010), and multidomain proteins are more common in eukaryotes (Ekman et al., 2005), this may to some extent reflect the fact that gene fusion events have often already occurred in eukaryotes, while prokaryotes are more likely to possess the prefusion forms. While there is little reason to think that the phenomenon of ordered assembly conservation would be unique to prokaryotes, we wished to confirm the relevance of our findings to eukaryotes. Therefore, we repeated our analyses using only protein complexes from eukaryotic species.

Using the same protocol described in the Methods, but limited to only eukaryotic complexes, we identified 11 nonredundant subunit pairs associated with gene fusion events (1azs A-B; 1be3 C-D; 1efv A-B; 1gaq A-B; 1q90 K-M; 1v54 A-B; 1v54 N-P; 2bfd C-D; 2fp4 A-B; 2ozl B-C; 3cx5 L-M).

With this smaller data set, we observed that 67% of gene fusion events in complexes with > 2 unique subunits conserved (dis)assembly, compared to only 9% expected by chance, which remained highly statistically significant ($p = 1 \times 10^{-4}$), despite the much smaller size of the data set. Furthermore, we observe real gene fusion events associated with 4/124 (3.2%) of the eukaryotic subunit pairs that would conserve (dis)assembly, compared to only 4/869 (0.5%) of pairs that would modify (dis)assembly (p = 0.01, Fisher's exact test). The interface reduction analysis also remained significant, with a mean reduction of 4.83 interfaces upon fusion, compared to 2.34 expected by chance ($p = 1 \times 10^{-4}$). Thus, although the frequency of gene fusion association with subunit pairs is much lower in eukaryotes, there is still strong selection for those fusion events that conserve (dis)assembly and simplify complex topology. Finally, in 4/4 cases, the fusion distance was shorter than the reverse distance (p = 0.1, binomial test).

The small number of gene fusion events associated with eukaryotic subunit pairs could also partially be due to the fact that there are far fewer eukaryotic than prokaryotic genomes in the STRING database, and therefore fusion events involving eukaryotes might be assigned a lower confidence score. Therefore, we lowered the STRING fusion threshold to 0.05 in order to identify more subunit pairs associated with putative fusion events. This increased our data set to 20 nonredundant eukaryotic subunit pairs (1azs A-B; 1be3 C-D; 1e1r A-G; 1efv A-B; 1gaq A-B; 1ikn B-C; 1n1j A-B; 1q90 K-M; 1tzy B-D; 1v54 A-B; 1v54 N-P; 2bfd C-D; 2fp4 A-B; 2h88 A-B; 2ozl B-C; 2puk A-B; 2y8l B-C; 3cx5 L-M; 3oee C-H; 3rnm A-C).

With this expanded eukaryotic data set, we observed that 41.7% of gene fusion events in complexes with > 2 unique subunits conserved (dis)assembly, compared to only 14.6% expected by chance ($p = 0.01$). We also observe real gene fusion events associated with 5/125 (4.0%) subunit pairs that would conserve (dis)assembly, compared to only 7/870 (0.8%) pairs that would modify (dis)assembly ($p = 0.01$, Fisher's exact test). There was a mean reduction of 3.33 interfaces upon fusion, compared to 2.16 expected by chance ($p = 0.001$). Finally, the fusion distance was shorter than the reverse distance in 9/12 cases ($p = 0.1$, binomial test).

Overall, these results demonstrate that the conclusions of our study are not limited to prokaryotic species and extend across the domains of life.

SUPPLEMENTAL REFERENCES

Benesch, J.L.P., and Ruotolo, B.T. (2011). Mass spectrometry: come of age for structural and dynamical biology. Curr. Opin. Struct. Biol. *21*, 641–649.

Bilder, P., Lightle, S., Bainbridge, G., Ohren, J., Finzel, B., Sun, F., Holley, S., Al-Kassim, L., Spessard, C., Melnick, M., et al. (2006). The structure of the carboxyltransferase component of acetyl-coA carboxylase reveals a zinc-binding motif unique to the bacterial enzyme. Biochemistry *45*, 1712–1722.

Brodkin, H.R., Novak, W.R.P., Milne, A.C., D'Aquino, J.A., Karabacak, N.M., Goldberg, I.G., Agar, J.N., Payne, M.S., Petsko, G.A., Ondrechen, M.J., and Ringe, D. (2011). Evidence of the participation of remote residues in the catalytic activity of Co-type nitrile hydratase from Pseudomonas putida. Biochemistry *50*, 4923–4935.

Carter, E.L., Tronrud, D.E., Taber, S.R., Karplus, P.A., and Hausinger, R.P. (2011). Iron-containing urease in a pathogenic bacterium. Proc. Natl. Acad. Sci. USA *108*, 13095–13099.

Ekman, D., Björklund, A.K., Frey-Skött, J., and Elofsson, A. (2005). Multi-domain proteins in the three kingdoms of life: orphan domains and other unassigned regions. J. Mol. Biol. *348*, 231–243.

Gruswitz, F., O'Connell, J., 3rd, and Stroud, R.M. (2007). Inhibitory complex of the transmembrane ammonia channel, AmtB, and the cytosolic regulatory protein, GlnK, at 1.96 A. Proc. Natl. Acad. Sci. USA *104*, 42–47.

Jansen, R., Greenbaum, D., and Gerstein, M. (2002). Relating whole-genome expression data with protein-protein interactions. Genome Res. *12*, 37–46.

Jin, L., Stec, B., Lipscomb, W.N., and Kantrowitz, E.R. (1999). Insights into the mechanisms of catalysis and heterotropic regulation of Escherichia coli aspartate transcarbamoylase based upon a structure of the enzyme complexed with the bisubstrate analogue N-phosphonacetyl-L-aspartate at 2.1 A. Proteins *37*, 729–742.

Kim, J., and Raushel, F.M. (2001). Allosteric control of the oligomerization of carbamoyl phosphate synthetase from Escherichia coli. Biochemistry *40*, 11030–11036.

Krissinel, E., and Henrick, K. (2007). Inference of macromolecular assemblies from crystalline state. J. Mol. Biol. *372*, 774–797.

Kulik, V., Hartmann, E., Weyand, M., Frey, M., Gierl, A., Niks, D., Dunn, M.F., and Schlichting, I. (2005). On the structural basis of the catalytic mechanism and the regulation of the alpha subunit of tryptophan synthase from Salmonella typhimurium and BX1 from maize, two evolutionarily related enzymes. J. Mol. Biol. *352*, 608–620.

Mack, E. (1925). Average cross-sectional areas of molecules by gaseous diffusion methods. J. Am. Chem. Soc. *47*, 2468–2482.

Pinkse, M.W.H., Maier, C.S., Kim, J.-I., Oh, B.-H., and Heck, A.J.R. (2003). Macromolecular assembly of Helicobacter pylori urease investigated by mass spectrometry. J. Mass Spectrom. *38*, 315–320.

Spraggon, G., Kim, C., Nguyen-Huu, X., Yee, M.C., Yanofsky, C., and Mills, S.E. (2001). The structures of anthranilate synthase of Serratia marcescens crystallized in the presence of (i) its substrates, chorismate and glutamine, and a product, glutamate, and (ii) its end-product inhibitor, L-tryptophan. Proc. Natl. Acad. Sci. USA *98*, 6021–6026.

Thoden, J.B., Wesenberg, G., Raushel, F.M., and Holden, H.M. (1999). Carbamoyl phosphate synthetase: closure of the B-domain as a result of nucleotide binding. Biochemistry *38*, 2347–2357.

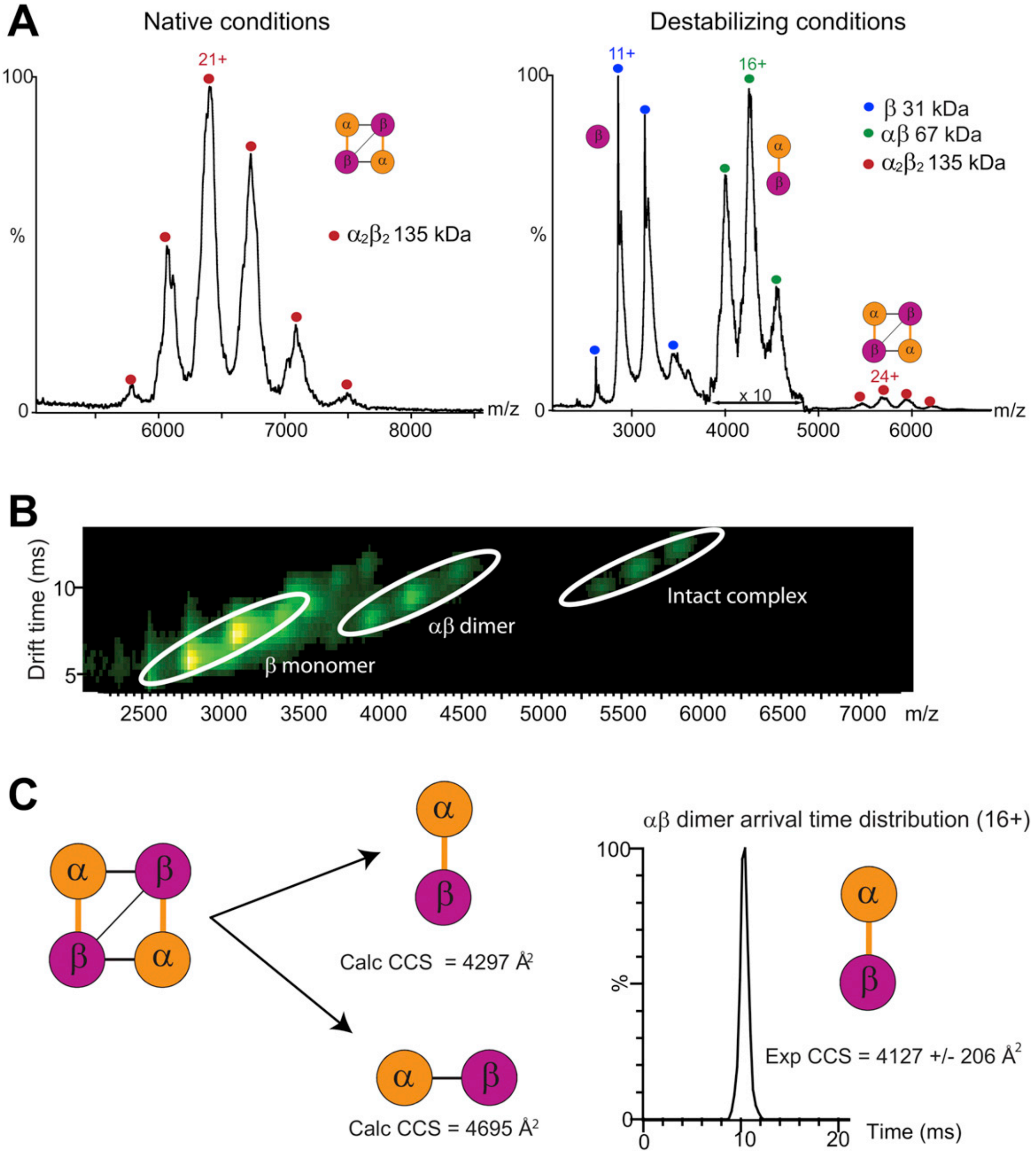

Figure S1. Acetyl-CoA Carboxylase Carboxyltransferase Experiments, Related to Figure 2

(A) Spectra from nESI-MS experiments observed under native (500 mM ammonium acetate) and destabilizing (30% MeOH, pH 6) conditions. Charge state series corresponding to the intact complex (red), the αβ dimer (green) and the β monomer (blue) are marked.

(B) Ion mobility drift time versus *m/z* contour plot in 30% MeOH, pH 6.

(C) Two αβ subcomplexes representing two alternate (dis)assembly pathways are possible. Comparison of the experimental collision cross section (CCS) with theoretical CCS values for the two possible dimers suggests that the dimer preserves the larger interface (orange). CCS values were calculated from the crystal structure using a scaled projection approximation (PA) method (Mack, 1925; Benesch and Ruotolo, 2011). The ion mobility arrival time distribution for the 16+ ion is shown.

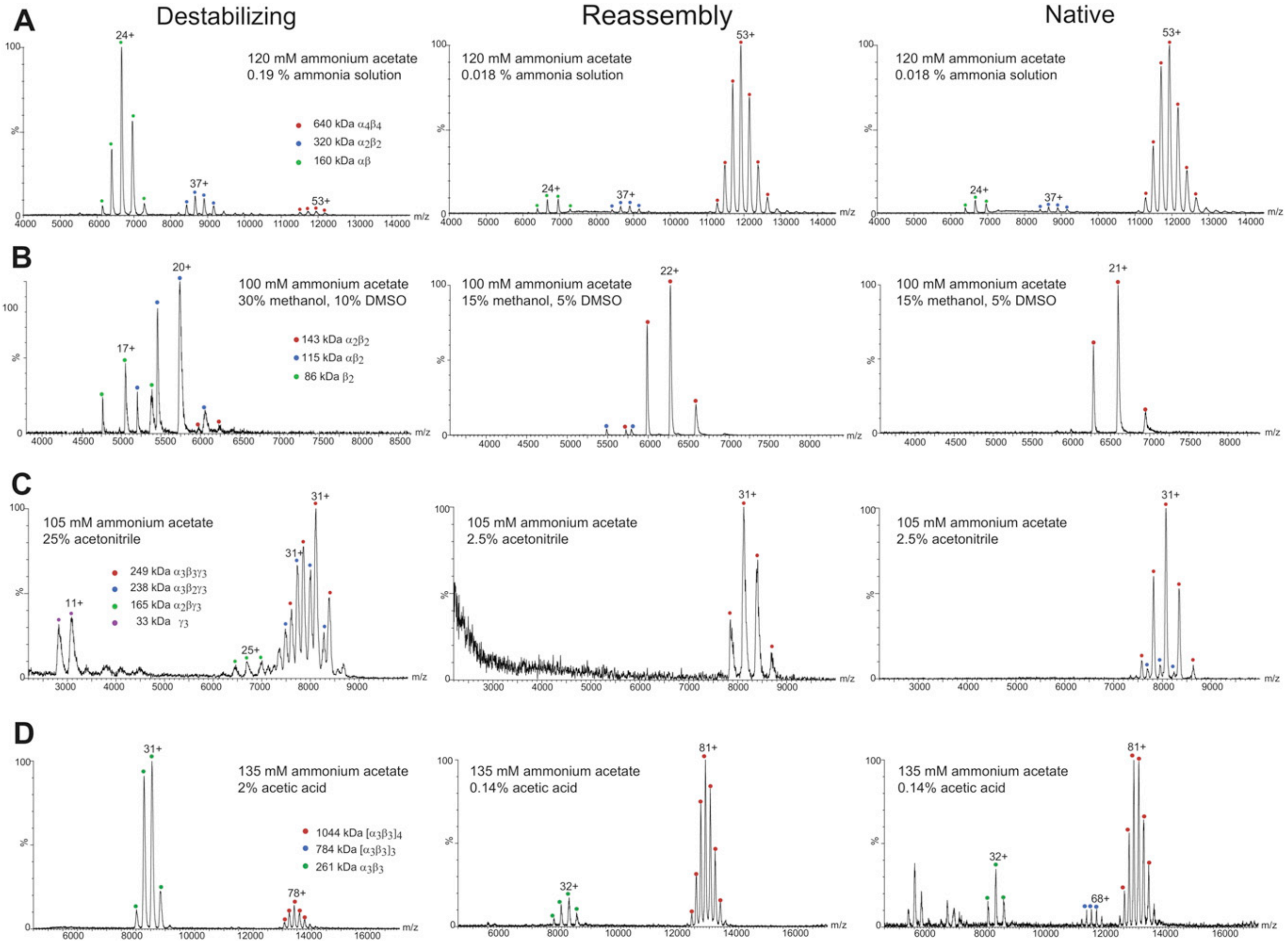

Figure S2. Reassembly Experiments, Related to Table 1

(A–D) Carbamoyl phosphate synthetase (A), tryptophan synthase (B), *K. aerogenes* urease (C), and *H. mustelae* urease (D). Mass spectra for each complex are presented under destabilizing conditions, after reassembly, and before (dis)assembly under native conditions.

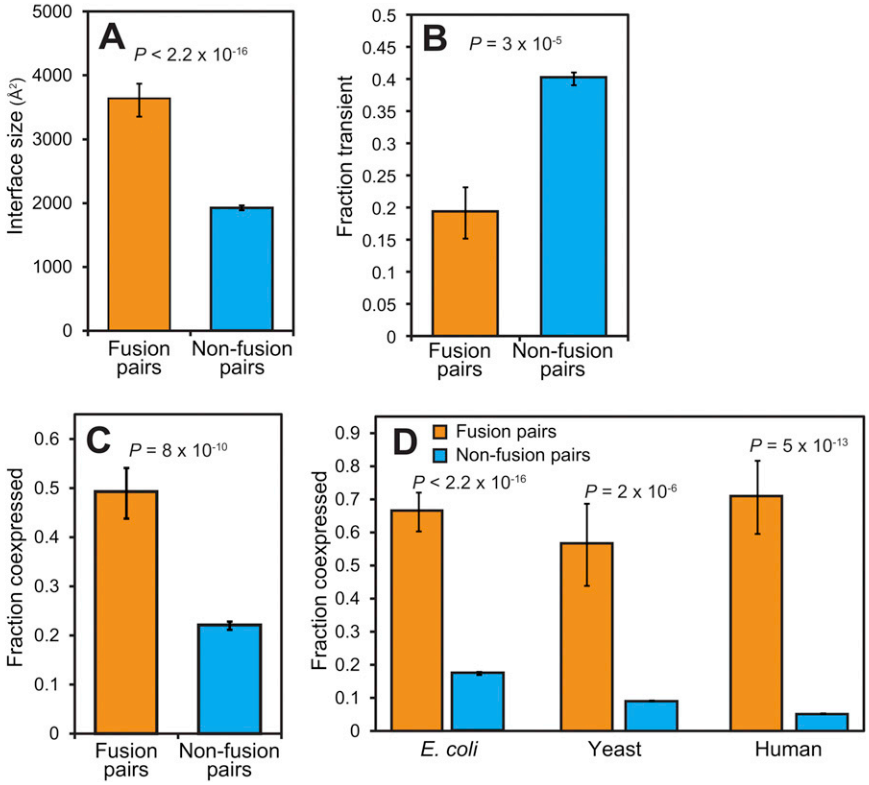

Figure S3. Preferential Fusion in Obligate Complexes, Related to Figure 3

(A–C) Comparison of (A) interface size, (B) fraction transient, and (C) coexpression in 94 fusion pairs and 2,449 nonfusion pairs from crystal structures.

(D) Comparison of coexpression in interacting pairs of proteins from high-throughput protein-protein interaction data for *E. coli* (65 fusion pairs and 6,422 nonfusion pairs), yeast (16 fusion pairs and 102 959 nonfusion pairs) and humans (17 fusion pairs and 59 703 nonfusion pairs). All error bars represent the SEM.
